# Feasibility of virtual reality and machine learning to assess personality traits in an organizational environment

**DOI:** 10.3389/fpsyg.2024.1342018

**Published:** 2024-07-24

**Authors:** Elena Parra Vargas, Lucia Amalia Carrasco-Ribelles, Javier Marin-Morales, Carla Ayuso Molina, Mariano Alcañiz Raya

**Affiliations:** ^1^Laboratory of Immersive Neurotechnologies (LabLENI) – Institute Human-Tech, Valencia, Spain; ^2^Instituto universitario de investigación en atención primaria “Jordi Gol”, Valencia, Spain

**Keywords:** personality traits, statistical machine learning, eye-tracking, virtual reality, big five traits, implicit measures

## Abstract

**Introduction:**

Personality plays a crucial role in shaping an individual’s interactions with the world. The Big Five personality traits are widely used frameworks that help describe people’s psychological behaviours. These traits predict how individuals behave within an organizational setting.

**Methods:**

In this article, we introduce a virtual reality (VR) strategy for relatively scoring an individual’s personality to evaluate the feasibility of predicting personality traits from implicit measures captured from users interacting in VR simulations of different organizational situations. Specifically, eye-tracking and decision-making patterns were used to classify individuals according to their level in each of the Big Five dimensions using statistical machine learning (ML) methods. The virtual environment was designed using an evidence-centered design approach.

**Results:**

The dimensions were assessed using NEO-FFI inventory. A random forest ML model provided 83% accuracy in predicting agreeableness. A *k*-nearest neighbour ML model provided 75%, 75%, and 77% accuracy in predicting openness, neuroticism, and conscientiousness, respectively. A support vector machine model provided 85% accuracy for predicting extraversion. These analyses indicated that the dimensions could be differentiated by eye-gaze patterns and behaviours during immersive VR.

**Discussion:**

Eye-tracking measures contributed more significantly to this differentiation than the behavioural metrics. Currently, we have obtained promising results with our group of participants, but to ensure the robustness and generalizability of our findings, it is imperative to replicate the study with a considerably larger sample. This study demonstrates the potential of VR and ML to recognize personality traits.

## Introduction

Personality traits reflect an individual’s characteristic patterns of thoughts, feelings, and behaviors (Brewer, 2019). An individual’s personality has been shown to affect various aspects, such as consumption habits, performance ability, interpersonal communication, mental health, and even political stance (Cai and Liu, 2022). Personality refers to cognitive and behavioral patterns that show stability over time and across situations (Cattell, 1943) therefore, it is reasonable to expect that personality traits influence personal values and attitudes, as Olver and Mooradian (2003) have demonstrated. Hence, personality is the key to understanding the adaptation of behavior (Martin et al., 2023).

The Big Five model proposed by Costa and McCrae provides a concise and comprehensive taxonomy of personality. Each personality dimension describes a broad domain of psychological functioning that is composed of a set of more specific and narrow traits (H. Zhao and Seibert, 2006), (Carducci et al., 2020). Five-factor theorists claim these factors, singly or together, can be found in virtually all personality instruments (Bayram and Aydemir, 2017). The Big Five model is widely used to analyze personality traits and behavior due to its impressive reliability and stability (Angelini, 2023). It is consistent across ages and cultures, and its predictive power has remained strong over time. These properties make the Big Five one of the most dependable and parsimonious models for explaining the complex interplay between personality traits and behavior (Roberts et al., 2006). This model determines five representative traits:

1. Extroversion indicates an individual’s comfort level with relationships. In this sense, individuals who stand out in this trait are usually sociable and assertive. While introverts tend to be reserved, shy, and quiet. This trait is characterized by the involvement of all team members, emphasizing the desire for openness and accessibility. It involves the collection of both professional and personal information to reach a consensus that is comfortable for all team members. It involves aspects such as empathy, consensus-seeking, and good communication within the team (Costa and McCrae, 1992).
2. Agreeableness indicates a person’s ability to adapt to others. Individuals with a high level of agreeableness are cooperative, warm, and trusting, while those with a low level are cold, unpleasant, and antagonistic (Costa and McCrae, 1992). This trait is associated with sympathy, empathy, trust, kindness, and altruism. Individuals who score high on agreeableness are often seen as friendly, kind, and helpful. They tend to be more cooperative, forgiving, and tolerant of others than those who score lower on agreeableness (Judge and Ilies, 2002).
3. Conscientiousness: This trait or dimension is based on self-control, not only of impulses but also in the planning, organization, and execution of tasks. Individuals with high levels in this dimension are usually responsible, organized, reliable, and persistent. In contrast, those with low levels are easily distracted, disorganized, and unreliable (Carducci et al., 2020).
4. Neuroticism reflects an individual’s ability to withstand stress. Individuals with low levels of emotional stability are calm and secure, while those with high levels are nervous, anxious, and insecure. They are characterized by either non-decision-making or delegating responsibility to other team members. This implies valuing opinions. A non-decision leads to the non-resolution of the problem (Costa and McCrae, 1992).
5. Openness: relates to an individual’s desire for novelty and ability to think creatively. Extremely open individuals are creative, curious, and artistically sensitive, while those at the other end of the spectrum are conventional and find comfort in the familiar. Individuals open to experience tend to be more efficient in solving problems (Robbins and Judge, 1996).

Therefore, personality traits are conceptualized as a set of stable individual differences in people’s motivational reactions to circumscribed classes of environmental stimuli (Bayram and Aydemir, 2017). Personality traits are stable patterns of behavior and therefore have an impact on decision-making style, which are situations that include the approach, reaction, and action of the individual who is about to make a decision (Van Scoy et al., 2023). The relationship between decision-making and personality has been studied in multiple areas such as stress (Buelow and Cayton, 2020), emotional intelligence (El Othman et al., 2020) and risky decision-making (Babakr and Fatahi, 2023), among others.

The investigation made by El Othman et al. (2020) showed a relationship between personality and decisions, where higher extroversion was associated with a less rational decision-making style, while higher agreeableness and conscientiousness were associated with a more rational decision-making style. By understanding an individual’s personality, organizations can create better working relationships and foster an environment of cooperation, trust, and productivity. Furthermore, an individual’s personality can affect how he/she communicates with their colleagues and deals with issues that arise. Bayram and Aydemir (2017), found in their study a relationship between decision-making styles and personality traits. On the one hand, they found a negative relationship between extraverted personalities and avoidant decision-making styles, additionally, they found positive correlations between rational, intuitive and dependent decision-making styles and agreeable personalities.

Decision-making in gamification involves considering diverse psychological constructs that can impact how individuals engage with and respond to game-based experiences. Some key psychological constructs to consider are motivation, engagement, emotion, personality, and cognitive processes (Almarshedi et al., 2017).

### Personality and organizational behavior

Organizational researchers have assembled an impressive body of knowledge about how personality relates to nearly all-important workplace behavioral and attitudinal criteria (Connelly et al., 2022). Angelini (2023) highlighted the relationship between personality traits and job burnout, finding that neuroticism correlated positively with burnout while the other personality traits correlated negatively with this domain.

Organizational health researchers, such as Wall and Berry (2007), found that personality traits were predictive of multiple organizational behaviors, with those high in conscientiousness and agreeableness exhibiting less counterproductive work behavior (e.g., theft, sabotage, withdrawal, production deviance, and abuse toward others) and those high in neuroticism being more prone to such behaviors (Wall and Berry, 2007). All these elements of organizational health had great relevance to the conscientiousness trait. In addition, another of the major personality traits, conscientiousness, was investigated within the framework of self-reports from a sample of 104 employees. This trait directly influenced performance within the context of organizations but was not related to well-being or perceptions within the work environment. Similarly, this trait reduced the impact of role clarity, reduced psychological distress, and increased job satisfaction.

The five-factor model has provided a useful taxonomy for studying job performance and leadership (Heller et al., 2002). In this regard, the study focuses on personality traits associated with organizational dynamics. Consistent with this, knowing personality can predict the pattern of human decision-making (Ju et al., 2016). In the study of Laguía et al. (2024), personality traits were found relevant predictors of job crafting. Job crafting is understood as an individually-driven work design process that refers to self-initiated, proactive strategies to change the characteristics of one’s job to better align with personal needs, goals, and skills. They found a positive relationship between extraversion, agreeableness, conscientiousness, openness and job crafting.

By recognizing the strengths and weaknesses of different personality types, managers can better manage teams and ensure that each employee feels valued and respected. Additionally, employers can use personality assessments to identify potential growth and development areas for their employees. Ultimately, leveraging the power of personality traits to create a positive work environment can lead to increased job satisfaction, improved team morale, and higher productivity (Manosalvas Vaca, 2017).

Validated psychological questionnaires that determine the values of model traits are traditionally used for this purpose. However, the fixed and lengthy nature of such questionnaires makes them impractical for many applications (Berkovsky et al., 2019). Personality, analyzed through the prism of the Big Five model, has been related to and studied in various areas such as relationships, emotional expression (Ruan et al., 2020), healthy behavior patterns (Carver and Scheier, 1982), leadership (Strang, 2004), and organizations (Miller et al., 1999).

Questionnaires and self-reporting are the traditional measures used to evaluate leadership style, personality, and organizational effectiveness, among others. These measures are effective but have some limitations due to the need for active user participation. For example, individuals may not always be accurate in their self-assessment, and their responses may be influenced by factors such as social desirability bias or response style. There are various old and recent studies indicating that self-report measures of personality appear susceptible to biased responses, especially when administered in competitive environments (Barrick and Mount, 1996; Ones et al., 1996; Hirsh et al., 2008). The feasibility of obtaining a user’s personality through self-reporting is very low for large-scale measurement (Cai and Liu, 2022). From the standpoint of ecological validity, they are decontextualized measures of real situations and do not elicit the same behavioral responses as in real life. Similarly, these self-report measures are limited by human perception, presenting as biases in social desirability and acquiescence, affecting the veracity of responses (Nederhof, 1985; Furnham, 1986; Grimm, 2010). We also highlight the growing concern in the contemporary literature about the effectiveness of such instruments and questionnaires (Fisher and Chaffee, 2018; Crawford and Kelder, 2019). Therefore, some researchers have required an analysis of leadership and personality from a different methodological perspective to identify halo effects, which do not capture these real behaviors (Baumeister et al., 2007) and threats to validity (Antonakis et al., 2010; Crawford and Kelder, 2019). These studies indicate that respondents often selectively enhance their positive traits while downplaying their negative ones. Therefore, it can be difficult to represent personality accurately when there is motivation for favorable self-presentation. One recent attempt has been made to address the problem of biased responses and the lack of success in detecting and controlling this tendency using a new comparative scaling method, in which each trait domain is scored relative to all the others rather than separately (Hirsh et al., 2008).

### Virtual reality and behavioral assessment

Virtual reality (VR) comprises a synthetic 3D environment in which users can interact naturally and realistically (Barnes, 2017; Parra et al., 2021). This technology can create a psychological phenomenon known as the sense of presence, which occurs when an individual feels as if they are present in a non-physical world. One benefit of using VR in experimental research in multiple sciences is that it is compatible with the collection of direct information from the user, both at the behavioral level (e.g., reactions, decision-making, and response times) and the neurophysiological level (e.g., brain activity, skin conductance, and cardiac variability) (Wirth et al., 2020). These latter responses, commonly recorded by external systems, provide valuable indirect sources of information related to human behavior in various facets, including leadership competencies (Marín-morales et al., 2018). The most commonly examined personality traits in virtual environments are absorption (Tellegen and Atkinson, 1974), mental imagination (Sheehan, 1967), locus of control (Rotter, 1966), dissociation (Bernstein and Putnam, 1986), and the five-factor model, which comprises several personality traits (Narooi and Karazee, 2015; Khatri et al., 2022).

According to experiences in many experimental studies, such as Neguț et al. (2016), a VR system should provide a realistic sense of immersion in the virtual world. These studies showed that using VR measures for assessment had high ecological validity since they allow functional abilities to be assessed in real-life situations. The most critical factor in this respect is social interaction, whose credibility is based mainly on social factors and emotional behavior when human interactions occur (Wang et al., 2010).

VR offers a unique environment that allows researchers to simulate different scenarios and measure individuals’ reactions in a controlled and safe setting. Overall, measuring psychological dimensions with VR can revolutionize the field of psychology by providing a more comprehensive understanding of human behavior and emotions. Wirth et al. (2020) measured the psychological dimension by developing a VR scenario to assess the Big Five personality traits (openness, conscientiousness, extraversion, agreeableness, and neuroticism) in team athletes. Their study found that the VR scenario effectively assessed the Big Five personality traits in team athletes and provided a more immersive and realistic assessment than traditional personality tests. Another example is the study by Gorini et al. (2011), which found that individuals with greater extraversion reported greater enjoyment and immersion in VR games, while individuals with greater agreeableness reported lower aggression in a VR fighting game. Some of the most notable studies on this subject have found that an individual’s personality is a major factor in determining their effectiveness within an organization. Studies have found that individuals with a well-defined sense of self-awareness, a strong sense of responsibility, and a strong commitment to their jobs are more likely to be effective in their roles (Boyatzis et al., 2000). De-Juan-Ripoll et al. (2021) and Khatri et al. (2022) found that VR could be used to identify personality traits based on consumer behavior. Physiological responses, self-reported anxiety and perceived risk measures were reliable trait indicators. In addition, Parra et al. (2021), developed a VR scenario to assess leadership skills and found that it effectively provided a more immersive and realistic environment for assessment. These findings suggest that combining VR technology with organizational neuroscience techniques could be used in leadership training and development.

In the field of personality analysis and VR, some studies have highlighted the relevance of avatars within the environment (Matamala-Gomez et al., 2019). These studies have identified particular personality traits that modify user behavior within a virtual environment, suggesting the possibility of predicting these traits based on user behaviors and relationships with avatars (i.e., how they answer and look at them) (Berkovsky et al., 2019). Specific personality traits, measured by their corresponding self-report questionnaires, are correlated with the user-perceived sense of presence using one of the various existing self-reported presence measures (Katifori et al., 2022). In addition, they allow the integration and collection of other implicit measures, such as brain activity, skin conductance, heart rate variability, and eye tracking. These measures provide valuable indirect sources of information related to the implicit correlations between organizational behaviors (Marín-Morales et al., 2018; Parra et al., 2021) and personality (Gawronski and De Houwer, 2014; Berkovsky et al., 2019). Additionally, VR provides a controlled and repeatable environment, allowing for more accurate and detailed data collection. In addition, virtual agents in VR can provide a more immersive and personalized experience for participants (Giglioli et al., 2021). Therefore, using ET (eye tracking) and decision-making in VR with virtual agents can be a powerful tool for measuring personality and better-understanding individuals’ cognitive and emotional processes (Parra et al., 2022).

While personality dimensions feature prominently in organizational behavior, little is known about how these traits can be predicted by implicit measures such as visual behavior and decision-making in realistic situations (Roberts et al., 2006). To overcome these limitations, advances in immersive VR technologies combined with implicit measures, such as behavioral decision-making, gaze patterns, and statistical machine learning (ML) techniques, have enabled the creation of similar virtual experiences to real ones. Therefore, they can better identify implicit behaviors and recognize behavioral styles more ecologically.

### Decision-making behaviors and eye-gaze patterns as implicit VR measures

The implicit measures aim to collect psychological attributes without requiring users to report a subjective assessment of them. The interactions of the users with the virtual environment can also be studied by analyzing their gaze movements (Parra et al., 2022), heart rate variability, and the skin galvanic response (Chicchi Giglioli et al., 2021).

Personality traits can also affect the autonomic nervous system and, in turn, the bodily and physiological responses that are determined by it (Vinciarelli and Mohammadi, 2014). Therefore, since eye movements and visual behavior are also implicit signals of the autonomic nervous system, they have been widely used to detect conscious and unconscious activities. Characteristics of this visual behavior have been defined as reliable indicators for cognitive strategies and attention (Raptis et al., 2017), cognitive load (Chen et al., 2022), and lie detection (Cipresso and Riva, 2016).

ET can provide useful information about an individual’s cognitive style, emotional state, and preferences. It has been used to identify emotions such as happiness, sadness, and surprise. It can also be used to study how individuals view different types of visual information, such as images, text, and videos. Ultimately, eye tracking can aid in understanding an individual’s personality and behavior (Berkovsky et al., 2019).

In the personality domain, evidence shows an association between visual patterns, facial features, and personality factors (Gavrilescu and Vizireanu, 2019). Early research on personality and visual behavior was directed at establishing the association between eye contact, gaze aversion, and sociability (Giglioli et al., 2021; Chen and Haga, 2022; Lamb et al., 2022). Berkovsky et al. (2019) predicted personality traits associated with the Big Five dimensions by analyzing subjects’ visual behavior and gaze patterns while observing images and videos with different emotional content. This study demonstrated that personality traits could be precisely determined by analyzing this type of unconscious behavior, showing the differences between subjects with different levels of the Big Five dimensions.

It has been recognized that the ease of use and affordability of eye-tracking equipment offer “unique and relatively unhindered insights into perceptual, cognitive, motivational, and/or affective processes underlying human behavior”(Ashby et al., 2016). Eye movements offer a unique window into the unobserved perceptual, cognitive, and evaluative processes of individuals engaged in decision-making tasks (Wedel et al., 2022). Glöckner and Herbold (2011) found that longer fixations were associated with deeper processing, such as careful consideration of information, while shorter fixations were associated with more superficial processing levels. This information is acquired by observing and paying attention to behaviors and facial expressions, which allow the detection of complex mental states, such as intentions, thoughts, beliefs, emotions, and desires of those around (Balconi and Canavesio, 2014). Therefore, through gaze, individuals attempt to accurately assess the motivations, intentions, and emotions to anticipate the behavior of another and to amend their own decisions and actions accordingly (Berchio et al., 2019).

Eye-tracking methods provide insights into the cognitive processes involved in behavioral decision-making that are not otherwise easily obtained (Parra et al., 2022). The eye movement and choice converge, suggesting that eye movement during decision-making reflects individual differences in social preference (Ashby et al., 2016). The dual processing model indicates that certain decision-making provokes responses from two separate but competitive psychological processing systems. One system involves automatical processes, which are fast, parallel, and effortless, and require minimal cognitive effort. In contrast, the other system involves controlled processes that are conceptualized to be slow, serial, and effortful, and require an individual’s complete attentional resources (Maie and Godfroid, 2022). Personality may be understood as the result of the typical functioning (across time and situations) of both types of processes.

Isaacowitz (2005) showed that implicit personality measures effectively assess individual differences in personality and behavior. By observing eye movements, their study found that optimists spent less time inspecting negative emotional stimuli than pessimists, and that extroverts tended to fixate on positive stimuli for longer than introverts. This finding suggests that implicit measures can be used to accurately measure individual personality traits and behaviors. In addition, Ju et al. (2016) demonstrated that psychopathology was negatively correlated to prosocial personality. This finding shows that personality differences can predict intuitive decision-making and that such a process can be studied in controlled immersive VR simulations.

This experience is difficult or impossible to achieve in laboratory settings since multi-sensory laboratory stimulation does not provide VR’s complete and immersive contextual experience. A review of social cognitive neuroscience and VR found that this technology was effective for affective induction, social psychology, and neuropsychological assessment (Parsons and Rizzo, 2008).

### Machine learning

ML is a scientific discipline within artificial intelligence that designs and develops algorithms that allow computers to develop behaviors based on empirical data, recognize hidden patterns, and use them to make predictions (Mikalef et al., 2018). Research has shown that ML approaches can have higher predictive validity than traditional self-report measures and questionnaires (Kern et al., 2016; Kosinski et al., 2016; Zhao et al., 2022). ML models have been used to predict individuals’ Big Five personality traits from diverse data sources, including digital footprints on social media platforms (Kosinski et al., 2013). It is becoming increasingly clear that ML also has the potential to transform research and assessment in personality psychology (Stachl et al., 2020).

Recently, a growing number of researchers have noted how ML techniques applied to big data can be used to study individuals’ behaviors in the workplace (George et al., 2014) or measuring executive personality (Hrazdil et al., 2020). Indeed, ML has been used to evaluate candidates (Faliagka et al., 2012), identify traits defining the leadership role (Ju et al., 2016; Doornenbal et al., 2022), and measure personality traits in executives (Wille et al., 2018). However, it has also been used to study and predict soft skills in organizations, such as the communication skills of a job candidate (Suen et al., 2020), and to evaluate workers’ soft skills based on behavioral signals, such as gaze and facial expressions (Muralidhar et al., 2018). However, using ML to predict interpersonal skills has mainly been in the clinical setting since it has been used more often in clinical psychology (Blease et al., 2021; Parra et al., 2022) than in organizational situations (Chicchi Giglioli et al., 2021). For example, ML has been used to assess therapists’ performance in specific essential competencies, such as empathy level (Gibson et al., 2022; Parra et al., 2022) based on implicit measurements collected within virtual environments.

This study aimed to recognize the Big Five personality dimensions (neuroticism, agreeableness, extraversion, openness, and conscientiousness) in individuals exposed to a 3D virtual environment simulating social interactions in the workplace. Visual behavior and decision-making were used as implicit measures. In addition, ML methods were used to analyze the implicit measures and explore whether it was possible to predict levels of personality traits and identify parameters that best discriminate between them.

This study’s main hypotheses were:

*H1:* Eye tracking is a useful implicit measure for assessing psychological behaviors in subjects within an effective context (a serious game in a VR context).

*H2:* The subjects’ decision-making during the experience would predict the Big Five personality traits.

*H3:* The virtual context helps to predict and modify psychological behaviors, which themselves help to predict personality traits.

## Methodology

This study investigates a comprehensive experiment aimed at measuring and developing a predictive behavioral model. The main objective is to assess several variables, including personality, attachment, and soft skills. Through a rigorous scientific approach, the aim is to establish meaningful relationships and build a theoretical framework to understand and predict human behavior more accurately. The interconnection and analysis of these variables constitute the methodological basis for achieving the objectives proposed in the context of this experiment. Therefore, the experimental procedure of this article is the same as the following Parra et al. (2022) and Parra et al. (2022).

In this context, the general objective of the research line focuses on the construction of a generalist behavioral model. Therefore, specific data related to the traits considered to be fundamental in user behavior will be presented. The collection and detailed analysis of this information will provide a solid basis for the formulation of a model that aims to comprehensively understand human behavior which studies the general behavior of people especially in the context of simulated social interactions in virtual environments., such as Peysakhovich and Naecker (2017) that in their study incorporate ML to the behavioral science of ambiguity aversion, or Pan et al. (2018), in which explains the importance of VR to study human social interaction. This approach seeks not only to identify individual patterns but also to establish meaningful connections between key variables that influence participants’ behavior.

### Participants

The study sample comprised 83 subjects, of which 32 were women and 51 were men (mean age = 42). All subjects were Caucasian, Spanish nationals, and Spanish-speaking. Being over 18 years old was the only inclusion criterion for participation in this study. Individuals were excluded if they had any mental disorder or were taking medication that affected their cognitive and mental functions.

The subjects’ levels of personality traits were determined using the NEO Five-Factor inventory (NEO-FFI) questionnaire, which was aimed to classify them into high and low neuroticism, extraversion, openness, agreeableness, and conscientiousness. A complete representation of each of the Big Five personality traits was obtained based on the questionnaire responses.

All subjects were interviewed, and asked how many hours a week they played video games; those who played ≤1 h a week were considered low, and those who played ≥3 h a week were considered high.

All participants provided their written consent to participate in this study. This study was conducted according to the 1964 Declaration of Helsinki and was approved by the Ethics Committee at the Polytechnic University of Valencia, Spain.

### Personality assessment scales

Subjects completed the NEO-FFI questionnaire for personality assessment. Developed by Costa and McCrae (1992), it comprises 60 items that operationalize the five major personality dimensions in the five-factor model (Costa and McCrae, 1992). Items in the NEO-FFI are empirically based and systemically sample the full range of personality traits. Items in the NEO-FFI are answered on a five-point scale ranging from (1) strongly disagree to (5) strongly agree, and scales are balanced to control for agreement. The NEO-FFI takes approximately 10 min to complete (Costa and McCrae, 1992).

### Virtual environment description

The evidence-centered design (ECD) guidelines were followed to create a valid measure to obtain reliable results from the VR experience. ECD is a framework used to guide the design and development of assessments, which starts to collect valid evidence from the beginning of the test creation process (Arieli-Attali et al., 2019). ECD involves evaluating the framework by collecting implicit measures, such as eye gaze patterns, as demonstrated by Hoppe et al. (2018). ECD-based technologies have been utilized for stealth assessment methods, providing valid and reliable frameworks for test design.

Originally developed in the education field to enhance the validity and reliability of test measures for students, ECD considers evaluations as arguments based on evidence. This means actions that allow observation of what students say or do at a specific moment, enabling inference about their knowledge, abilities, or achievements (Mislevy et al., 2003).

Following these guidelines, a story narrative was designed with scenes set in different office environments. The virtual environment comprised four situations with the same organization. This study involved participants experiencing a virtual office environment. They were given two interactive tasks-chatting with co-workers and answering emails to help them better understand the work environment. The chatting with co-workers activity evaluated the participants’ decisions in a chat group with their colleagues. They used a virtual keyboard to chat about topics such as internet jokes, humorous images, and personal problems. Examples included a recruitment task with blurred images and a video with audio issues to emphasize the importance of paying attention to verbal and non-verbal cues. The decision-making regarding how often the participants opened, answered, and sent messages was also evaluated. Upon completing the tasks, the participants were provided with their performance results and asked to provide feedback.

**Figure 1:**
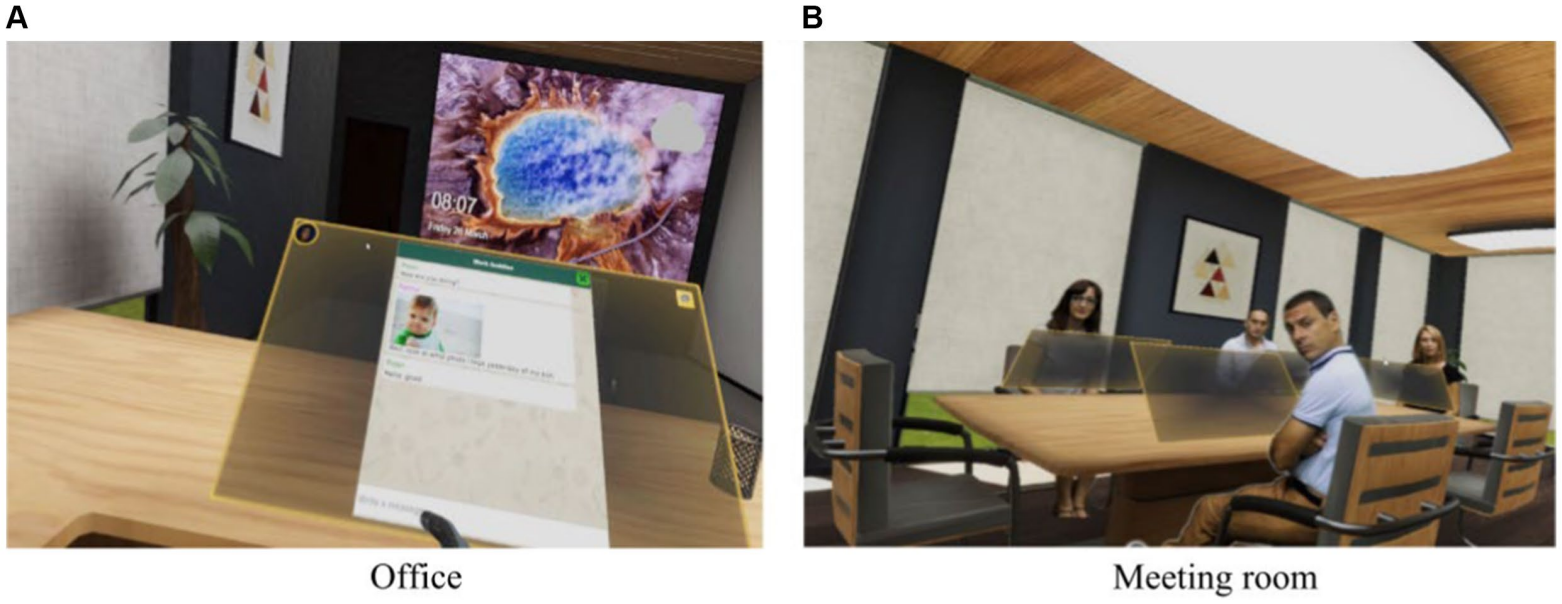
Scenarios of the virtual reality. **(A)** office and **(B)** meeting room. Reproduced from Parra Vargas et al. (2022).

Decision-making analysis and eye-tracking data took particular importance in the data collection since four scenarios were created to measure the same construct in this way. Specifically, the four scenarios were (Figure 1):

1. Office environment: four virtual agents with different personalities and behaviors were described. They were presented in a group meeting scenario where participants had to decide based on their decision-making style. Four different decision-making styles were used in a meeting room scenario. Style 1 involves cooperation, interest in the welfare of others, and emotional responses. Style 2 involves not making decisions unless the opinion of others is known due to an excessive concern for rejection. Style 3 is characterized by rapid and rigid responses, minimal trust, and dislike when the rest of the team disagrees. Style 4 is defined by a complete lack of interest in others and a lack of cooperation and support. Each decision style was characterized by different attitudes, such as cooperation, minimal trust in others, and a complete lack of interest in others. Participants were asked to make decisions as a group and individually, with mini-games being implemented as filler tasks.
2. Meeting room environment: The meeting participants returned to the office and were asked to rate their behavior and performance in the group chat and problem-solving tasks. They were then shown various mini-games and encouraged to select which ones they wanted to play.
3. Back in the office: the participants at the meeting were asked to evaluate their behavior and performance in the group chat and problem-solving tasks. They were then given a selection of mini-games to play and asked to rate their performance and provide their opinion of the game. The virtual environment was designed to encourage empathy-related behaviors using avatars with different personality traits and tasks related to personal and work decisions.

Overall, the virtual environment was designed to stimulate behaviors related to personality traits with the help of avatars and various decision-making tasks.

**Figure 2:**
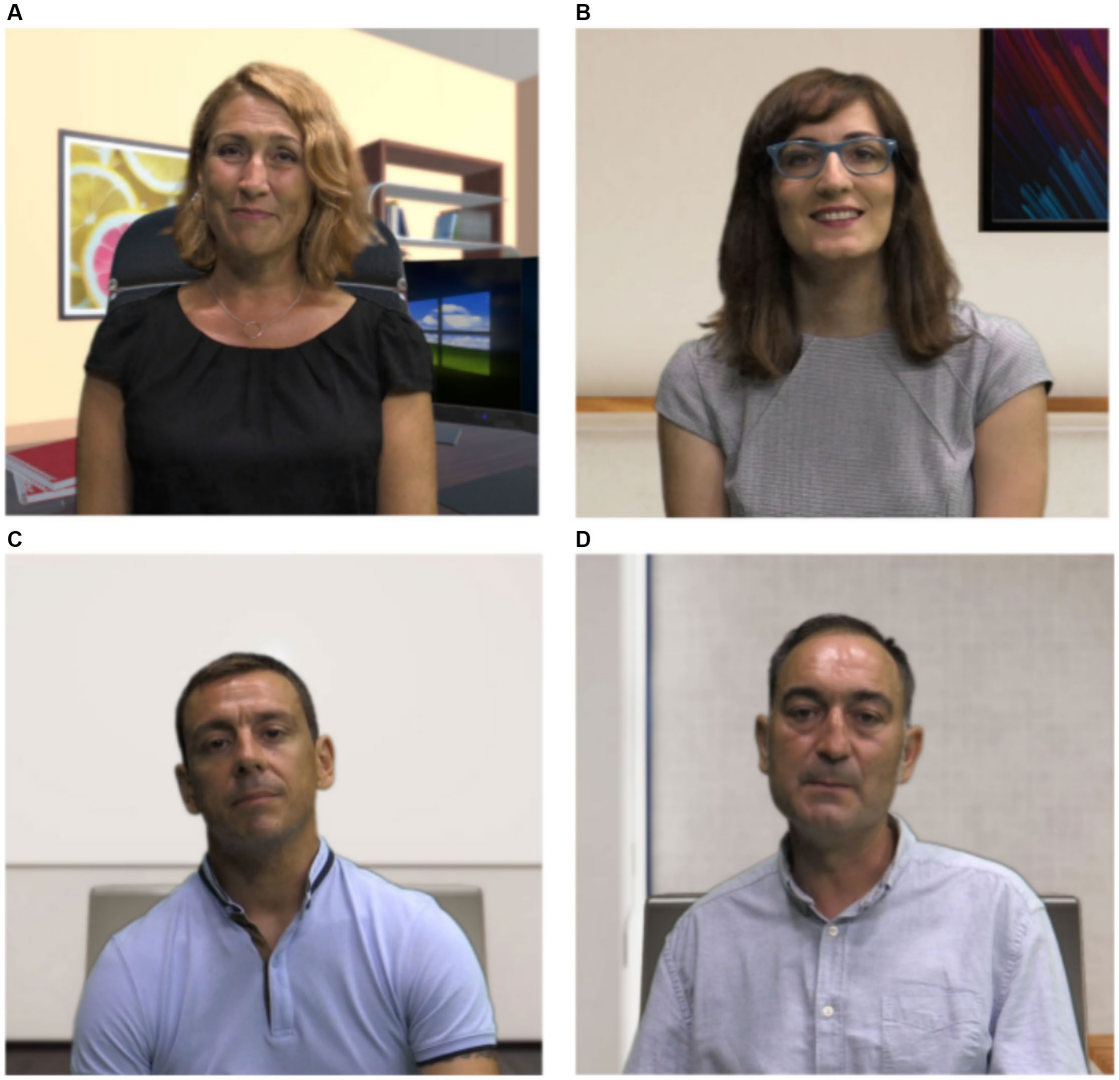
Virtual agents. **(A)** virtual agent character, **(B)** organiser agent, **(C)** passive agent, and **(D)** logical agent. Reproduced from Parra Vargas et al. (2022).

**Figure 3:**
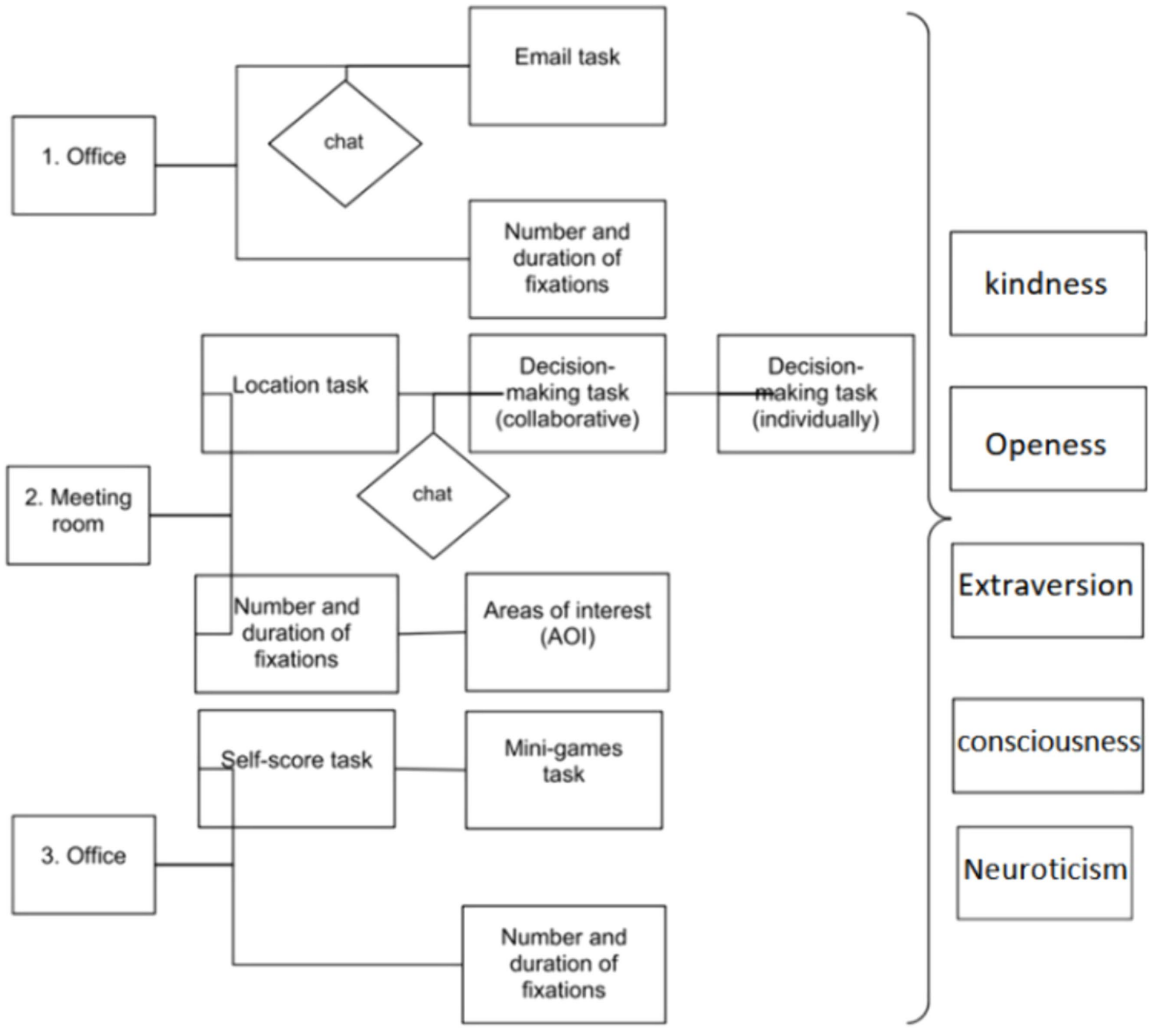
Virtual reality outline. Adapted from Parra Vargas et al. (2022).

In the meeting room, they shared a table with other co-workers, who encouraged them to interact and make decisions. This meeting room scenario involved four adult virtual agents (two women and two men) designed with different personality traits and soft skills, to collect information from interactions with different contextual, social, and emotional characteristics (Figure 2). Specifically, one of the characters was defined as an organizer, another as emotional-interpersonal, another as logical, and the last as non-interventional (described in Appendix A1):

In each meeting situation, 2–3 discussion points were presented for resolution among the team members. These situations involved interactions between virtual agents sought the user’s opinion, who was asked to freely express their views verbally and to select the option most aligned with their opinion. Several response alternatives representative of different decision-making styles were presented to do this (see Figure 3). According to Scott and Bruce (1995), individuals generally have different levels of all styles, although one is usually dominant, and this profile tends to be reasonably stable over time (Scott and Bruce, 1995; Bruine de Bruin et al., 2007). Each possible decision was developed using a systematic method based on the Big Five personality styles: extraversion, neuroticism, kindness, conscientiousness, and openness to experience (see Appendix A2).

### Experimental procedure

To determine the personality traits within the sample, the participants completed the NEO-FFI questionnaire. They also completed a short demographic questionnaire to collect data related to age, sex, and job position. After completing them, participants visited the laboratory to complete the experimental testing through VR. The experimental phase comprised a single 1.5-h session in which the participants experienced a simulation in an immersive VR environment. The first 2 min of the experience showed a brief tutorial explaining how to use the virtual environment. The eye-tracking application was started manually at the beginning of the session, and calibration was conducted once it was placed on the participant. After these steps, the virtual environment simulation began, and the user was immersed in the first scene of the first situation (the office). Once the first situation was completed, the next situation began until the entire experience was completed (four situations in total).

The visual attention was measured using the HTC Vive Pro Eye head-mounted display, with a combined resolution of 2,880 × 1,600 pixels (1,440 × 1,600 per eye), a 110° field of view, and a refresh rate of 90 Hz. VR was applied using a 17.3” MSI GE75 Raider 9SF-1204XES laptop (Intel i7-9750H processor, 32 GB of RAM, 1 TB NVMe PCIe Gen3x4 SSD, and GeForce RTX 2070 graphics card with 8 GB of GDDR6 SDRAM).

The VR system was developed using Unity 5.5 1f1 software, applying C# pro254 programming language with the Visual Studio tool.

## Neo-FFI

### Data processing

Data were obtained from three different sources: the answers to the NEO-FFI questionnaire, the behavioral data (i.e., decisions made by participants in the VR experience), and eye-tracking data (i.e., sight fixations). The raw behavioral and eye-tracking data were transformed into a set of variables. The behavioral data comprised 63 variables (Table 1). If the participant did not complete the entire VR experience or all mini-games assessing self-efficacy, the missing values were filled with a category indicating this situation. The eye-tracking data comprised 110 variables (Table 2).

**Table 1:** Description of the variables obtained from the decisions made during the VR experience.

Big Five trait	Description
Conscientiousness	ResponseClick. Answers provided by the participant
Neuroticism	OrientationClick: each time the participant answers an email
Agreeableness	TestUserClick: each time the participant plays a game
Openness to experience	Location the participant chooses on the meeting table (1 × Situation + Mode)
Extraversion	Use of the messaging app: usefulness score given (13), number of times opened, number of messages sent, and real interaction (times open – messages sent)

**Table 2:** Description of the variables obtained from the eye-tracking data.

Variable	Description
Fixations	Mean number (and standard deviation) of fixations performed per situation and in total during the whole SG.
Sx_Participant_VirtualAgent	Per situation (4×), the average time (s) the participant looks at each virtual agent (6×) while the participant is speaking.
Participant_VirtualAgent	Over the entire experience, the average time (s) the participant spends talking to and looking at each virtual agent (6×).
Sx_VirtualAgentA_VirtualAgentB	Per situation (4×), the average time (s) the participant looks at virtual agent B while virtual agent A is speaking (6×).
VirtualAgentA_VirtualAgentB	Over the entire experience, the average time (s) the participant looks at virtual agent B while virtual agent A is speaking.

The environment was considered another possible area to look at while speaking, so the time spent looking at it was also calculated since it was itself a virtual agent.

### Statistical analysis

Three participants who did not respond to the NEO-FFI questionnaire were excluded from the analysis. A multivariate outlier analysis considering the questionnaire’s four dimensions was performed (Filzmoser, 2004). In this outlier detection method, the distance between participants was calculated by considering all questionnaire subscales and estimating the probability of this distance belonging to a Chi-square distribution. If this probability was <0.01, the participant’s scores were defined as outliers. Four participants were considered outliers based on this approach and were excluded from further analysis. Finally, 77 participants were considered.

The mean, median, minimum, maximum, standard deviation and interquartile range were used to describe the NEO-FFI scores. The normality of the scores was assessed using the Shapiro–Wilk test. Statistical significance was defined as a *p* < 0.05.

### Machine learning

ML models were trained to predict the participants’ scores on the NEO-FFI questionnaire from their behavior in the VR experience. To train these models, one was created per subscale, the scores were categorized as high or low according to the median of each subscale.

First, feature selection was performed using a backward sequential wrapper to reduce the number of features. This method starts by building a model based on a particular ML algorithm with all available features and measuring its performance. Then, at each step, a feature is removed, the model is re-trained, and its performance is measured. The feature whose removal increased the performance measure (i.e., Cohen’s Kappa) the most was removed from the set of features used in the next step. The process stops after several steps in which the performance metric does not vary by >0.01.

Different ML algorithms were used to obtain the best feature set: random forest, support vector machine (SVM), Naïve Bayes, XGBoost, and *k*-nearest neighbor (kNN). These algorithms used the default hyperparameters defined in the *mlr* package (v2.14.0) (Bischl et al., 2016). After obtaining the optimal feature set for each ML algorithm, the model was trained and validated, and its metrics (i.e., accuracy, Cohen’s Kappa, sensitivity) [true positive rate (TPR)], and specificity [true negative rate (TNR)] were calculated. Both steps used repeated cross-validation (fivefold, four times), so the validation metrics correspond to the mean value across 20 repetitions. The same folds were used to validate all algorithms. The information from 10 randomly selected participants was excluded from this building model process and was used only as a test set. The statistical and ML analyses were performed in the R statistical software (version 3.6.1).

## Results

### NEOFFI scores description

Table 3 describes the scores for the NEO-FFI subscales. Participants scored 16.65 ± 6.92, 31.82 ± 7.17, 28.48 ± 6.08, 26.75 ± 4.93, and 32.94 ± 5.8 in neuroticism, extroversion, openness, and responsability, respectively (mean ± standard deviation; Shapiro–Wilk *p* > 0.05). The median ± interquartile range of kindness was 26.75 ± 4.93 (Shapiro–Wilk <0.05). Once categorized, 56, 52, 56, 53, and 53% of the participants had scored high on the neuroticism, extraversion, openness, kindness, and responsibility subscales, respectively.

**Table 3:** Summary of the participants’ scores for each NEO-FFI subscale.

Subscale	Mean	Median	Standard deviation	Interquartile range	Minimum	Maximum	Shapiro–Wilk normality test *p*-value	High score (*N*)	Low score (*N*)
Neuroticism	16.65	16	6.92	10	4	33	0.14	43	34
Extraversion	31.82	32	7.17	10	13	45	0.29	40	37
Openness	28.48	28	6.08	8	12	45	0.97	43	34
Kindness	26.75	27	4.93	6	9	40	0.04	41	36
Responsibility	32.94	33	5.80	8	21	47	0.62	41	36

The last two columns show the number of participants in each category after discretizing the scores according to the median value for each subscale.

### NEO-FFI recognition models

Table 4 shows the metrics and features selected in the best ML models for each NEO-FFI subscale. The best model for predicting kindness was a random forest with 17 features (58.82% from the eye-tracking data and 41.18% from behavioral data), achieving 80 and 83% accuracy in the validation and test sets, respectively. The best model for predicting openness was a kNN with 19 features (57.90% from the eye-tracking data and 42.10% from the behavioral data), achieving 70 and 75% accuracy in the validation and test sets, respectively. The best model for predicting extraversion was an SVM with 20 features (50% from the eye-tracking data and 50% from the behavioral data), achieving 80 and 85% accuracy in the validation and test sets, respectively. The best model for predicting neuroticism was a kNN with 29 features (51.72% from the eye-tracking data and 48.28% from the behavioral data), achieving 74 and 75% accuracy in the validation and test sets, respectively. Finally, the best model for predicting responsibility was a kNN with 12 features (91.67% from the eye-tracking data and 8.33% from the behavioral data), achieving 86 and 77% accuracy in the validation and test sets, respectively. All models had high TPRs and TNRs, with little difference between them in the validation set, except for the neuroticism model. The results in Table 4 show a TNR of neuroticism of 0.6 and a TPR of 0.84. Therefore, the data is consistent with the interpretation of neuroticism in predicting high neurotic behavior. However, is not as accurate as in TPN when the person has low neurotic behavior. Consequently, the prediction of low neurotic behaviors is not so accurately perceived. Due to this, the differences in the model of neuroticism are higher than in the other models.

**Table 4:** Metrics of the best model for each NEO-FFI subscale in the validation and test sets.

Subscale	Model	Features (*n*)			Validation set					Test set				
		Eye-tracking	Behavioral	Total	Accuracy	Kappa	AUC	TPR	TNR	Accuracy	Kappa	AUC	TPR	TNR
Kindness	Random forest	10	7	17	0.80	0.59	0.84	0.83	0.77	0.83	0.67	0.94	1.00	0.67
Openness	kNN	11	8	19	0.70	0.37	0.70	0.71	0.69	0.75	0.47	0.71	0.86	0.60
Extraversion	SVM	10	10	20	0.80	0.58	0.82	0.79	0.79	0.85	0.70	0.86	0.71	1.00
Neuroticism	kNN	15	14	29	0.74	0.42	0.68	0.84	0.60	0.75	0.50	0.78	0.83	0.67
Conscientiousness	kNN	11	1	12	0.86	0.71	0.87	0.86	0.88	0.77	0.54	0.93	0.71	0.83

The number of variables each model used was divided according to their source (i.e., eye-tracking or behavioral data). The values shown per metric in the validation set are the mean values of the cross-validation iterations. TPR and TNR represent the true positive and true negative rates, respectively.

## Discussion

This study used a VR strategy for relatively scoring an individual’s personality to measures captured from users interacting in VR simulations of different organizational situations. The results showed that it is possible to identify the Big Five personality traits in a virtual 3D environment. Behavioral (i.e., decision-making) and attentional (eye-tracking) data were measured in real-time during the VR experience and then modeled using ML methods to predict personality traits. Furthermore, the ML methods could accurately predict the levels of personality traits, suggesting that the implicit measures obtained in the VR environment are valid personality indicators. Additionally, this study identified the parameters that best discriminate between the Big Five personality traits, which can be used to inform the design of VR environments in personality assessment.

The ECD metrics enabled the building of ML models based on the collected eye-tracking and behavioral decision-making data from the VR experience. This process allowed for determining the variables that best predicted and characterized each personality dimension and analyzing the trait frequency distribution. The analysis results highlighted the predictive power of the variables, suggesting new possibilities for using VR to assess personality. By combining VR with behavioral and attentional measures, this multi-faceted approach offers a deeper understanding of the psychological construct and its manifestations. Moreover, it supports the ecological validity of self-report measures since it captures behavioral decision-making data in scenarios that imitate actual management situations.

This study enables our understanding of the practical implications and benefits of using implicit measures, specifically eye-tracking and decision-making data. Using ML models provided us behavioral estimates for VR experience participants’ NEO-FFI scores. These results showed good accuracy for every one of the Big Five personality traits, which was best for kindness. Based on these findings, both implicit measures enabled the measurement of personality traits more ecologically, offering valid data during the VR experience.

### Personality traits and implicit measures

The results collected by measuring psychological behaviors and collecting implicit measures allow us to understand the relationship between personality traits and implicit measures, the psychological behaviors linked to personality are more intuitive, and emotional. These behaviors do not need to be thought of, people behave related to some patterns established, and those patterns are the personality traits (Bayram et al., 2017).

Personality traits can be predicted by eye gaze and psychological behaviors, it seems in the results that people behave related to their personality traits. The data supported our research questions: Eye tracking is a useful implicit measure for assessing psychological behaviors in subjects within an effective context (a serious game in a VR context). All models were measured by both of them (eye tracking and psychological behavioral). However, the results showed better predictivity by the measures taken with eye tracking, the data taken were represented between 50% and 91.67%, conscientiousness is the trait with the highest punctuation. Meanwhile, the behavioral data were represented between 48.28 and 8.33%, neuroticism was the trait with the best score collected with behavioral data.

Therefore, eye tracking understood as implicit measures have a more relevant and distinctive role in predicting the different personality traits than the psychological behaviors did during the VR immersion. This could be explained by the idea proposed by Morewedge and Kahneman (2010) which describes two systems of thinking: system 1 is more intuitive and automatic, this system generates impressions, intuitions and response tendencies. Meanwhile, system 2 involucrate the process more logical and rationally. Due to this argumentation and the results, it seems that the eye gaze pattern could be part system one of Kahneman’s decision making. Therefore, through gaze, individuals attempt to accurately assess the motivations, intentions and emotions to anticipate the behaviors of others and to amend their own decisions and actions accordingly (Singer et al., 2004). When eye movements and decisions align, the movements reflect individual differences in social preferences.

Considering that behavioral data add value to the ML model, it supports the idea that personality traits are response tendencies, more intuitive and automatic. These automatic ways of behave have repercussions in all fields of work, especially in management environments in which decisions have to be made under uncertainty, risk and stress, personality is important due to the predictivity of the behavior based on the individual’s personality (Judge et al., 2002).

According to the metrics derived from the ML model, the virtual environment exhibits the potential for effectively monitoring behavioral aspects such as eye-gaze patterns and decision-making processes. This, in turn, allows for the categorization of participants based on their varying personality traits. Nevertheless, our findings indicate that the identification of personality traits is more accurately discernible through eye-tracking measures rather than through the analysis of decision-making behaviors. Eye tracking-related personality traits were significantly selected more frequently for all personality traits, being extroversion the only trait that gets the same predictivity with both metrics. This could be explained because extroversion traits are characterized by the involvement of all team members, emphasizing the desire for openness and accessibility, leadinging to more similar behaviors and eye patterns among them. Moreover, extraversion and neuroticism produced the best models in terms of absolute accuracy and similarity between the results in the validation and test set. According to a study by Kang et al. (2023), compared the personality differences between employees, supervisors, managers, and entrepreneurs, the results showed that entrepreneurs and managers exhibit lower neuroticism comparing the personality differences between employees supervisors, managers, and entrepreneurs the results showed that entrepreneurs and managers exhibit lower neuroticism compared to employees. The Big Five fundamental personality traits demonstrate general stability over time. Hence, many behaviors associated with these traits can be acquired through experience and conscious effort. Our findings demonstrate that by relating behavioral measures such as ET to personality traits, the detection of personality traits is effective, since it provides accuracy. Additionally, decision-making helps to refine the model. This is important for future applications of the relationship of personality with other variables such as leadership or attachment.

Measuring implicit personality traits showed that the soft skills-based transformational leadership model effectively improved the on-duty soft skills (leaders’ soft skills related to adaptability, communication, teamwork, and problem-solving) covering adaptability, communication, teamwork, and problem-solving (Morozevich et al., 2022). Academics such as Hautala (2006) and Kang et al. (2023) have investigated the personality and effectiveness of leadership to identify those traits that best fit professionals with high levels of effectiveness in leading teams in organizational settings. Expanding knowledge of the neuropsychological aspects responsible for the behaviors of individuals can form the basis for modifying and training effective leadership behaviors via interventions promoting them. Therefore, this assessment tool could be used again after the training phase to check training efficacy. The methodology could be applied in assessing other physiological constructs in clinical and organizational areas.

Furthermore, research has shown a correlation between implicit measures and soft skills understood as communication, listening, time management, problem-solving, leadership, and empathy (Fazio and Olson, 2003). Therefore, since soft skills are essential for success in the workplace, understanding how implicit measures can be used to assess and predict them can benefit employers. Additionally, research has shown that implicit measures can provide insight into an individual’s creativity, problem-solving, communication, and other important soft skills.

Eye movement parameters were extensively used to detect conscious and unconscious activities (Berkovsky et al., 2019; Evin et al., 2022). Complex features, such as gaze patterns and scan paths, were found to be reliable indicators of cognitive strategies and attention (Raptis et al., 2017). In the personality domain, early research established the links between eye contact, gaze aversion, and sociability (Libby and Yaklevich, 1973). With the advent of eye-tracking technologies, features derived from saccades, eye fixations, and pupils were found to be associated with personality traits (Berkovsky et al., 2019), therefore it’s demonstrated that eye-tracking data is a valuable implicit measure to predict personality traits. This behavioral prediction of the personality is helpful to establish other categories such as leadership, referring to leadership styles as a ‘pattern of behaviors’(Fischer and Sitkin, 2023). These definitions of leadership styles are quite similar to definitions of personality traits, which also concern stable patterns of behavior. Hence the relationship between leadership behaviors and personality traits is related to the behavioral characteristics of the person (ET patterns). Studies have shown that eye tracking measures an individual’s psychological behavior and can be a reliable predictor of leadership styles (Gerpott et al., 2018).

## Conclusion

This study focused on VR and implicit measures, such as visual behavior, as a paradigm for measuring personality traits. The procedure followed the ECD method, exposing participants to a 3D environment that simulated social interactions in the workplace. ML methods were used to analyze the implicit measures to explore whether it was possible to recognize levels of personality traits and identify the parameters that best discriminate between them.

With this experimental procedure, what we have achieved is the creation of a pilot of a behavioral prediction model based on personality traits, by combining eye tracking and the collection of decision-making behaviors. This pilot represents a promising first step toward a more comprehensive and sophisticated methodology that aims to unravel the relationship between underlying aspects of personality and how they could be predicted by using observed behavior.

This study’s main contribution is that it provides a multi-method approach that enables the capture, analysis, and recognition of personality traits. It also created an effective virtual context that evoked the behaviors necessary to assess individuals’ personality traits.

Overall, the research suggests that VR and ML can be valuable tools for assessing and predicting personality traits, with potential applications in areas such as personalized training recruitment and psychological assessment.

### Limitations and future directions

In this study, we identified some limitations that could be helpful for future research on personality and the organizational field. First, the small number of participants (*n* = 83) restricted our ability to generalize the results. Therefore, the test set for the ML-based models was also small. Second, since we built the high and low target variables based on the mean or median values of the responses in the study, they may not be extrapolated to the rest of the population. Therefore, both limitations compromise the generalizability of the theory. Additionally, we only recorded eye tracking for monitoring implicit measures; voice and heart rate variability could provide more information.

The aim of this study is not to supplant conventional selection tools like questionnaires or interviews, but rather to examine the viability of developing an assessment for personality traits that is more ecologically valid. By incorporating behavioral measures, we aim to replicate the results obtained from the NEO-FFI and create a more comprehensive evaluation of individuals’ personality traits.

One of the future research directions that looms as crucial in the field of the study at hand is the expansion of our sample. Currently, we have obtained promising results with our group of participants, but to ensure the robustness and generalizability of our findings, it is imperative to replicate the study with a considerably larger sample. This will allow us not only to confirm the validity and replicability of our findings but also to explore in greater depth the subtleties and variations that may arise in different demographic subgroups. In addition, a larger sample will provide us with the opportunity to conduct more detailed and advanced analyses, which will ultimately enrich our understanding of this evolving area of research.

In terms of future avenues, this research can establish a foundation for exploring psychological concepts such as personality traits by leveraging innovative technology like virtual reality combined with implicit measures and machine learning to make insightful predictions. To enhance the credibility of data, it is advisable to augment future studies by expanding the participant pool and incorporating expert evaluations to gauge the levels of personality traits exhibited by individuals. However, further studies should be conducted to validate our results and to analyze more deeply and comprehensively the predictive capacity of these personality traits for an employee’s effectiveness in an organizational context. Additionally, studies should be conducted to analyze the impact of other variables, such as motivation, professional development, and leadership, on the employee’s effectiveness. Furthermore, research should be conducted to analyze how personality traits could be used to improve the functioning of a team and organizational well-being in general.

Regarding future directions, this study can serve as a basis for studying psychological constructs, including personality, using a novel technology (e.g., VR with implicit measures and ML) to make predictions. Moreover, this study could lead to training and improving leadership skills, enhancing the work environment. It is recommended that future studies increase the number of participants and include more implicit measures presented by participants to improve data validity.

## Data availability statement

The raw data supporting the conclusions of this article will be made available by the authors, without undue reservation.

## Ethics statement

The studies involving humans were approved by the Ethics Committee of the Polytechnic University of Valencia (protocol number: P01_08_07_20). The studies were conducted in accordance with the local legislation and institutional requirements. The participants provided their written informed consent to participate in this study. Written informed consent was obtained from the individual(s) for the publication of any potentially identifiable images or data included in this article.

## Author contributions

EV: Conceptualization, Investigation, Methodology, Project administration, Supervision, Validation, Writing – original draft, Writing – review & editing. LC: Data curation, Formal Analysis, Methodology, Software, Writing – review & editing. JM: Conceptualization, Data curation, Formal analysis, Investigation, Methodology, Software, Supervision, Writing – review & editing. CA: Writing – original draft, Writing – review & editing. MA: Funding acquisition, Project administration, Resources, Supervision, Writing – review & editing.

## Appendix A1

1. The virtual organizer agent (female) is characterized by planned, sequential and structured thinking. Her role focuses on exposing the issues and leading the dialog. She is the one who decides what to do after hearing the opinions and counter-opinions of others but does not get involved either positively or negatively. However, this virtual agent tends to be open when promoting alternative decisions and proposing divergent decision paths.
2. The emotional-interpersonal or communicative virtual agent (female) is characterized by presenting empathic traits, interpersonal warmth, fluid communication, and holistic thinking. This agent talks about the topic to be discussed with confidence in herself and the team and encourages everyone to be part of reaching a consensual decision. She strives to understand the point of view and emotions of others. This character is approachable and sensitive to both internal and external problems. This virtual agent’s most outstanding traits were extraversion and agreeableness, both related to communication decision-making and challenging to differentiate in an organizational context.
3. The logical virtual agent (male) is characterized by presenting mathematical, technical, and analytical reasoning, with a tendency toward negativity. He does not present empathetic attitudes toward the rest of the characters; however, he presents distant, critical, and competitive behaviors. Additionally, this agent sets clear standards to follow and punishes any mistake. This virtual agent was characterized by great consciousness.
4. The last agent (male) is characterized by non-intervention. He avoids giving any feedback about his opinion. He leaves the decision to the team, so he trusts others’ points of view. However, he can be agitated depending on the situation without considering how this can affect others emotionally. This virtual agent was characterized by a neurotic personality.

## Appendix A2

The participant’s decision reflected the following behavioral styles:

Decision style 1: Cooperation was sought, reaching agreements among all members. Interest in the welfare of others was appreciated, and emotional responses were provided to demands. The following is an example: “We could focus on deciding who will be responsible. What do you think? Do you think we can distribute the tasks as I propose?”

Decision style 2: This style involved not making decisions unless the opinion of others was known, which was explained by excessive concern about rejection. Decisions showed high sensitivity to the emotions of others. However, extreme rejection concerns may leave few cognitive resources to understand such emotions. The following is an example: “Perhaps I have been here too short a time to be able to divide the tasks. I think it would be advisable for you to decide this time.”

Decision style 3: This style was characterized by rapid and rigid responses. Decisions reflected minimal trust in others and a dislike for when the rest of the team disagreed. Decisions did not reflect a willingness to understand or respond appropriately to the emotions of others. However, there was a great fear of rejection and a need for approval. The following is an example: “From my point of view, the best distribution is this.”

Decision style 4: This style was defined by a complete lack of interest in others and a lack of cooperation and support. No interest or concern for others was shown. There was no willingness to consider another individual’s perspective or share their emotions. The participants distanced themselves abruptly or stopped collaborating because they felt pressured. The following is an example: “Maybe we should go to the next point.
